# The CHILD safeguarding simulation study: **C**o-designed c**H**ild-centred **I**nterprofessional **L**earning through **D**ialogue for healthcare professionals

**DOI:** 10.1186/s41077-025-00403-w

**Published:** 2026-01-09

**Authors:** Michelle O’Toole, Walter Eppich, Clare Sullivan, Naoise Collins, Dani Hall, Aideen Walsh, Michelle Whelan, Claire Mulhall, Andrea Doyle

**Affiliations:** 1 RCSI SIM Centre for Simulation Education and Research, RCSI University of Medicine and Health Sciences, Dublin, Ireland; 2 Department of Medical Education and Collaborative Practice Centre, Faculty of Medicine, Dentistry and Health Sciences, University of Melbourne, Melbourne, Australia; 3 Department of Technology and Psychology, IADT, Institute of Art Design and Technology, Dublin, Ireland; 4 Pediatric Academic Health Science Centre, Children’s Health Ireland, Dublin, Ireland; 5 Laurels Clinic (Pediatric Forensic Medical Unit), Children’s Health Ireland, Dublin, Ireland; 6 Centre for Mastery: Personal, Professional & Academic Success (CoMPPAS), RCSI University of Medicine and Health Sciences, Dublin, Ireland

**Keywords:** Child safeguarding, Child protection, Interprofessional education, Interprofessional simulation, Post-registration/postgraduate learning, Co-design, Simulation-based education, Experiential learning

## Abstract

**Background:**

Globally, in excess of one billion children experience violence and abuse every year, leading to upwards of 40,000 deaths. Child safeguarding education typically occurs in professional silos across healthcare, often focusing on specific undergraduate competencies. In practice, however, child safeguarding requires a multi-professional approach, necessitating effective communication in emotionally charged contexts. To address these needs, we designed an interprofessional course using simulation-based education for experienced healthcare professionals working in the emergency department.

**Methods:**

On three occasions, we delivered an in-person, two-day course with 32 healthcare professionals from medicine, nursing, and social work. We collected data using multiple methods including participant demographics and child safeguarding experience (*n* = 32), observational field notes, individual semi-structured interviews (*n* = 14) and focus groups (*n* = 4). We analyzed the data using landscapes of practice theory as a sensitizing concept.

**Results:**

Using landscapes of practice theory, we deductively generated three key themes from our data: (1) collaborative learning, (2) the medium of language, and (3) creating a safe space. These themes encapsulate our participants’ experiences in navigating interprofessional learning within newly established teams, during simulated child safety scenarios in the emergency department. Findings also detail participants’ knowledge gains and confidence in reporting child safeguarding concerns.

**Conclusions:**

This co-designed interprofessional simulation-based child safeguarding course created space for learners to renegotiate safeguarding as a shared, interdependent responsibility. Authentic, emotionally charged scenarios in a psychologically safe environment helped participants tolerate uncertainty, rehearse reporting decisions, and develop a shared safeguarding lexicon. The resulting design principles may assist educators seeking to foreground psychological safety, authentic collaboration and the child’s voice in interprofessional safeguarding education.

**Supplementary Information:**

The online version contains supplementary material available at 10.1186/s41077-025-00403-w.

## Background

**Fig. 1:**
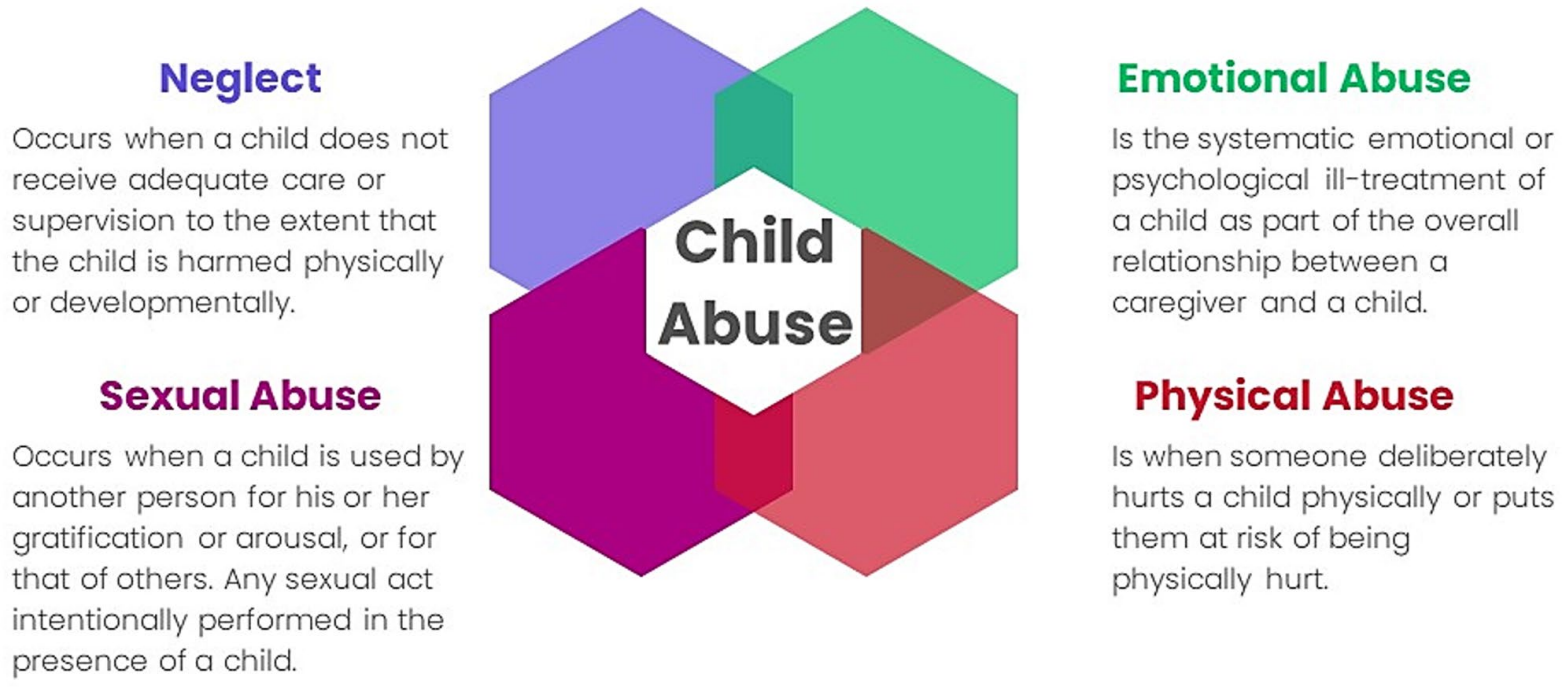
Four categories of child abuse

Worldwide, over 1 billion children per year experience violence and abuse, causing long-term emotional, social, and economic consequences, including over 40,000 deaths [1]. When healthcare professionals (HCPs) suspect child protection concerns, they must communicate these concerns with parents/caregivers empathetically and unambiguously. Within safeguarding frameworks, child abuse is conceptualized through four distinct yet interconnected categories [2]: neglect, emotional abuse, physical abuse, and sexual abuse (see Fig. 1). This categorization provides necessary structure for identification and intervention, also accounting for the stark reality that children often face multiple, overlapping forms of maltreatment. However, these sensitive situations challenge professionals as well as parents and caregivers, leading to additional anxiety in an already stressful situation. Evidence indicates that HCPs may not possess the requisite communication skills to effectively address concerns about potential signs of child abuse with parents [2–5]. These “difficult conversations” [4] present emotional difficulties for HCPs [6] and parents alike [7].

Child-safeguarding “…*is the action that is taken to promote the welfare of children and protect them from harm. While protecting children from abuse is one part of safeguarding, children and young people also need safeguarding in order for them to grow, develop and achieve their full potential*.” ( [7], p.6). These concerns also place unique demands on communication among multi-disciplinary and interprofessional teams. Indeed, even after children at-risk for abuse are identified, nearly all failures of child protection involve communication breakdowns between professionals [8]. These communication barriers contribute to underreporting of suspected child abuse concerns [2, 5]. Additionally, parents in these situations may be psychologically and physically exhausted; they may reject professional support and develop negative biases towards child welfare agencies, making effective social support even more important [9]. Thus, HCPs require not only training to improve the detection and prevention of child abuse [2], they must also communicate effectively with parents and caregivers of children at risk of abuse or neglect, all while sensitively managing difficult emotions and feelings. Further, these HCPs must communicate in ways that foster cooperation and engagement with both the HCPs and child welfare services.

The remit for child protection does not reside in one single agency. The scope of the problem places the onus on professionals across sectors and agencies to work together not only to recognize signs of potential abuse, but to ensure that individual cases traverse the various points within the system without falling through the cracks. At all stages in the process, high levels of professional awareness, sensitive and empathetic practice must be combined with effective communication skills and multi-agency working [10]. Only then will the system, meant to ensure child safety, both identify and deal with these difficult situations effectively, thus preventing additional harm and even preventable deaths.

Unfortunately, current child protection training is referred to as “atomistic” meaning it operates in isolation from other related components [11], typically in professional silos [12], viewing collaboration as a technical problem rectified through “best practice” guidelines or online modules [2]. These modules may provide the necessary knowledge related to child safety, yet they do not address the considerable team and communication skills required to ensure that children-at-risk and families successfully navigate a highly complex social system. This siloed approach also neglects unpredictable contextual situations, which may trigger conflict and mistrust within interprofessional teams and highlight the extremely social nature of team-based clinical practice. In child safeguarding, this team-based practice is essential for the interprofessional collaboration required for effective child safeguarding, highlighting the need for integrated approaches to interprofessional education (IPE) [8, 13–15]. An extensive literature base emphasizes the role of IPE in prelicensure health professionals [16]; unfortunately, this work often highlights approaches that bring together learners with little to no lived clinical experience and does not address the needs of practicing health professionals.

Multidisciplinary simulation-based education (SBE) curriculum design offers a potential approach to embed complex cases of child abuse into an interprofessional learning experience that fosters contextual collaborative skills in unpredictable environments. IPE creates positive interaction, encourages interprofessional collaboration and improves client care [17]. How to effectively design such interprofessional courses for a postgraduate audience remains less clear. Such a course would require the alignment of ‘*language*,* learning approaches and curriculum timetables and …people’* [18].

Child safeguarding is not simply a uni-professional area of knowledge [14]. This system involves a chain of professional practices including health and social care, education, and law. The whole system learns from the interplay between the knowledge and practices as professionals cross sectoral boundaries and interact with different professions [19]. To truly harness the collective expertise across these diverse professional boundaries, a co-design approach becomes essential; one that brings together professionals from all relevant disciplines as equal partners in the design process [20], ensuring that safeguarding systems are built upon integrated knowledge rather than aggregated individual perspectives [21]. Such an approach recognizes that effective child protection emerges not from the sum of separate professional contributions, but from the collaborative creation of shared understanding and coordinated practice [13]. By facilitating collaborative learning environments, professionals from different disciplines can explore their own and others’ roles and contributions to child safeguarding [22]. This cross-disciplinary perspective helps organizations understand how learning happens not just within communities, but across the boundaries between them, revealing opportunities for knowledge sharing and collaboration that might not be obvious when looking at individual communities in isolation [22].

This study aims to explore how a co-designed interprofessional simulation-based child safeguarding course can (a) prepare healthcare professionals to contribute to the care of children-at-risk through effective interprofessional teamwork and collaboration, and (b) advance the science of IPE by generating explicit design principles that create conditions conducive to cross-professional learning.

## Methods

### Study context

The study was conducted at the Royal College of Surgeons Ireland (RCSI), University of Medicine and Health Sciences, and received ethical approval from the RCSI research ethics committee (REC 202206004). Three iterations of the CHILD safeguarding simulation course were delivered and evaluated.

### Theoretical framing

A sociocultural perspective, namely landscapes of practice (LoP) theory, served as our theoretical framework for understanding learning within the child safeguarding courses [23, 24]. LoP acknowledges that learning is more than an individual process; learning is influenced by social and cultural practices of a particular community or group. Learning and knowledge creation are dynamic processes as individuals and communities interact. In the child safeguarding context, the care of these at-risk children demands collaboration and coordination between hospital-based practitioners and those in outpatient jurisdictions- paramedics, physicians, nurses and social workers to name a few. All of these professional groups represent distinct communities of practice (CoP). LoP builds on Lave and Wenger’s 1991 CoP model [25] and better addresses the complexities of learning in diverse and interdisciplinary contexts, such as the practice of child safeguarding. Ideally, the boundaries between CoP within a LoP are fluid and permeable, enabling the sharing of knowledge and resources across communities. The conditions that promote boundary spanning behaviors in child safeguarding are essential given the interprofessional nature and complexity of healthcare.

### Course design/description

We implemented and evaluated three iterations of the two-day, in person simulation-based interprofessional child safeguarding course. The course was situated within the specific context of an emergency department setting, where instances of child abuse and neglect often become apparent and demand an immediate interprofessional response.

Through an iterative and collaborative co-design approach, we recruited three pediatric professionals (doctor, nurse, and social worker) who served as co-faculty throughout the implementation. These individuals were experienced professionals from the national children’s hospital group in Ireland, Children’s Health Ireland, and the national child protection agency, Túsla. Prior to course implementation, we facilitated both scenario design and faculty development workshops, centred on the PEARLS model for debriefing [26], to prepare co-faculty and provide opportunities for practice. During the course, all simulation scenarios were debriefed by pairs of debriefers from different professions to ensure multi-professional perspectives were represented.

Seven simulated participants (SPs) were recruited to participate in the course. These SPs (members of a professional acting group with extensive experience in the RCSI teaching activities) portrayed simulated parents across the three course iterations. The SP group comprised one male and six females, aged between 26 and 55 years, with professional experience ranging from 5 to 30 years. All SPs completed a structured training program provided by RCSI SP educators, following the Association of SP Educators’ Standards of Best Practice [27], encompassing simulated role portrayal, healthcare consultations, and feedback provision. For this course, SPs received additional scenario-specific training, including access to scenario scripts and pre-briefing materials one week prior to each iteration. Detailed descriptions of the simulated scenarios, including learning outcomes, SP role portrayal guidance, and debriefing scripts for co-debriefers, are provided in Appendix A.

Given the sensitive and emotionally challenging course content, children and young people were not directly involved in development. Instead, we worked with an external advocacy agency, Empowering People in Care (EPIC), whose facilitators helped ensure the child’s perspective was embedded throughout and reinforced the importance of child-centred care.

The intervention is reported using the TIDier checklist (Appendix B) as well as aligning to healthcare simulation research reporting guidance [28].

### Participant recruitment

Participants included postgraduate HCPs who were actively working with children within the care pathway for at-risk populations in the Republic of Ireland. The course recruited professionals from four specific groups: pediatric nurses (N), pediatric doctors (Dr), pediatric medical social workers (MSW), and community child protection (Túsla) social workers (TSW). Eligible participants were professionally employed in health or social care disciplines that designated them as mandated persons for child safeguarding reporting, requiring them to previously complete online training in child protection from the Health Service Executive (HSE), the national public health and social care service. Participants were identified via the research team’s professional networks and invited to participate via email. The Participant Information Leaflet and consent form were provided: (a) by email at recruitment, allowing participants time to consider their participation, and (b) on the day of the intervention. Purposeful sampling ensured a balance from various professional groups. Researchers recruiting participants and collecting data were not involved in their employment or professional evaluation.

### Data collection

We used multiple methods for collecting data for this study, including participant demographics and child safeguarding experiences within the previous three months, observational field notes, followed by post-intervention semi-structured interviews and focus groups. The overarching research programme was informed by co-design workshops with multiple professionals involved in the chain of child safeguarding in Ireland.

Qualitative data was collected through multiple methods to capture participants’ learning experiences and decision-making processes. Post course semi-structured interviews (*n* = 14) and focus groups (*n* = 4, with 12 participants in total) explored participants’ perspectives on interprofessional collaboration, learning outcomes, and the application of knowledge gained through the simulation-based training (see Appendix C for interview guide). The interviews occurred online, 2–4 weeks post course completion, and lasted on average 30 min in duration, while focus groups took 45–60 min. Interview or focus group choice was determined by participant availability and logistical considerations.

### Data analysis

We used theoretical thematic analysis to analyse qualitative data from interviews supplemented by field observations [29]. The LoP framework served as a sensitizing concept to guide the deductive analysis, providing theoretical lenses through which interprofessional learning and boundary-crossing behaviors within the child safeguarding context could be examined. We used Nvivo to support data analysis.

### Reflexivity

Our team comprised various professional backgrounds, which contributed to our analytic framing. Five members of the research team had ‘insider status’ in the studied professional domains, so we reflected on our individual positionality during analytical team meetings [30]. MOT is a former first responder with responsibility for child safeguarding, and a health professions educator. WJE and DH are both pediatric emergency physicians, health professions educators with simulation expertise. AW is a pediatric nurse specialist with vast experience in child safeguarding in sexual assault treatment centers. MW is a social worker with a background in the national child protection agency. CS is a simulation researcher, with expertise in SP methodology. NC is a former primary school teacher, simulation researcher and lecturer in game design. CM is a geologist, a lecturer in simulation with SP methodology expertise and AJD is a medical physicist, health professions educator with SP methodology expertise.

## Results

### Participant demographics

**Fig. 2:**
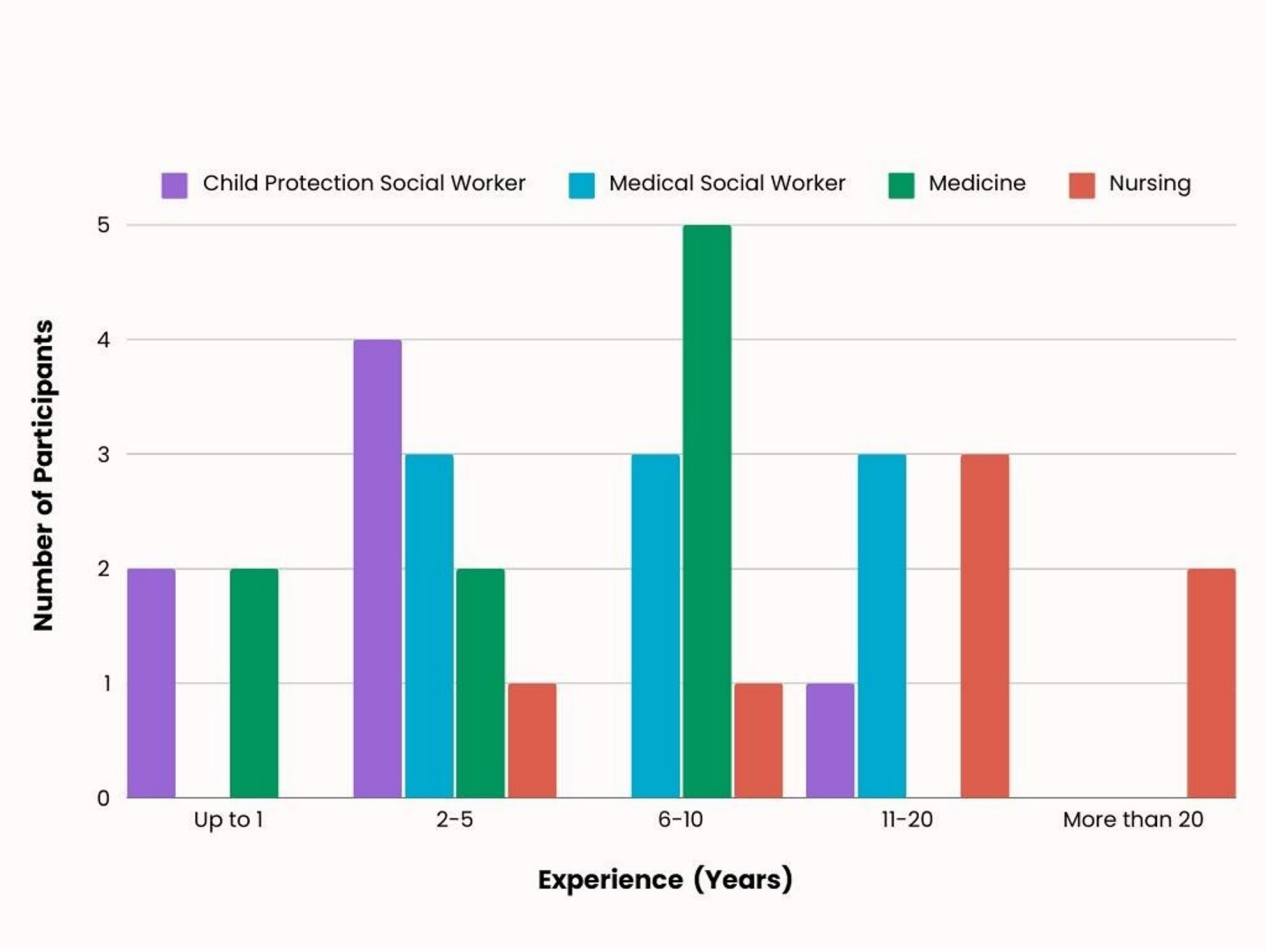
Participant experience in their professional role

Thirty-two participants (25 female, 7 male) in total participated across three iterations of the course: *n* = 12 (iteration one), *n* = 10 (iteration two), and *n* = 10 (iteration three). Participants included medical doctors and nurses working in pediatric context, as well as social workers from both the clinical context and the community child protection agency in Ireland, Túsla. Participants reported varying levels of experience in their professional role, from one year up to more than 20 years’ experience (See Fig. 2).

**Fig. 3:**
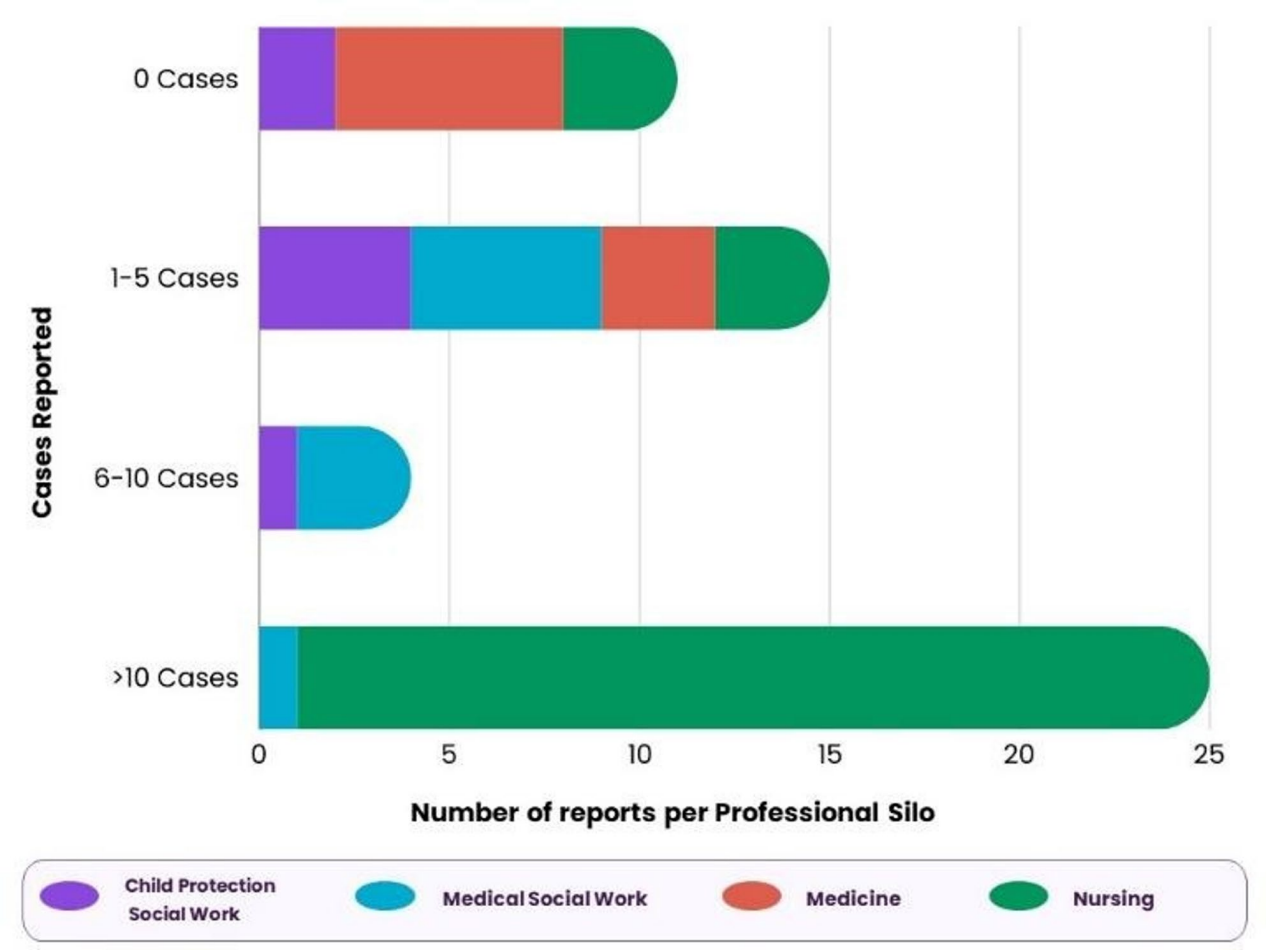
Number of cases reported by participants three months prior to training intervention

To understand participants’ familiarity with the process of reporting suspected child safeguarding concerns, we asked about the number of cases they reported in the three months prior to taking part in the training intervention (see Fig. 3). Participants, on average, reported up to 5 cases, however, one nurse reported 24 suspected cases of child abuse in the three months prior to the training.

**Fig. 4:**
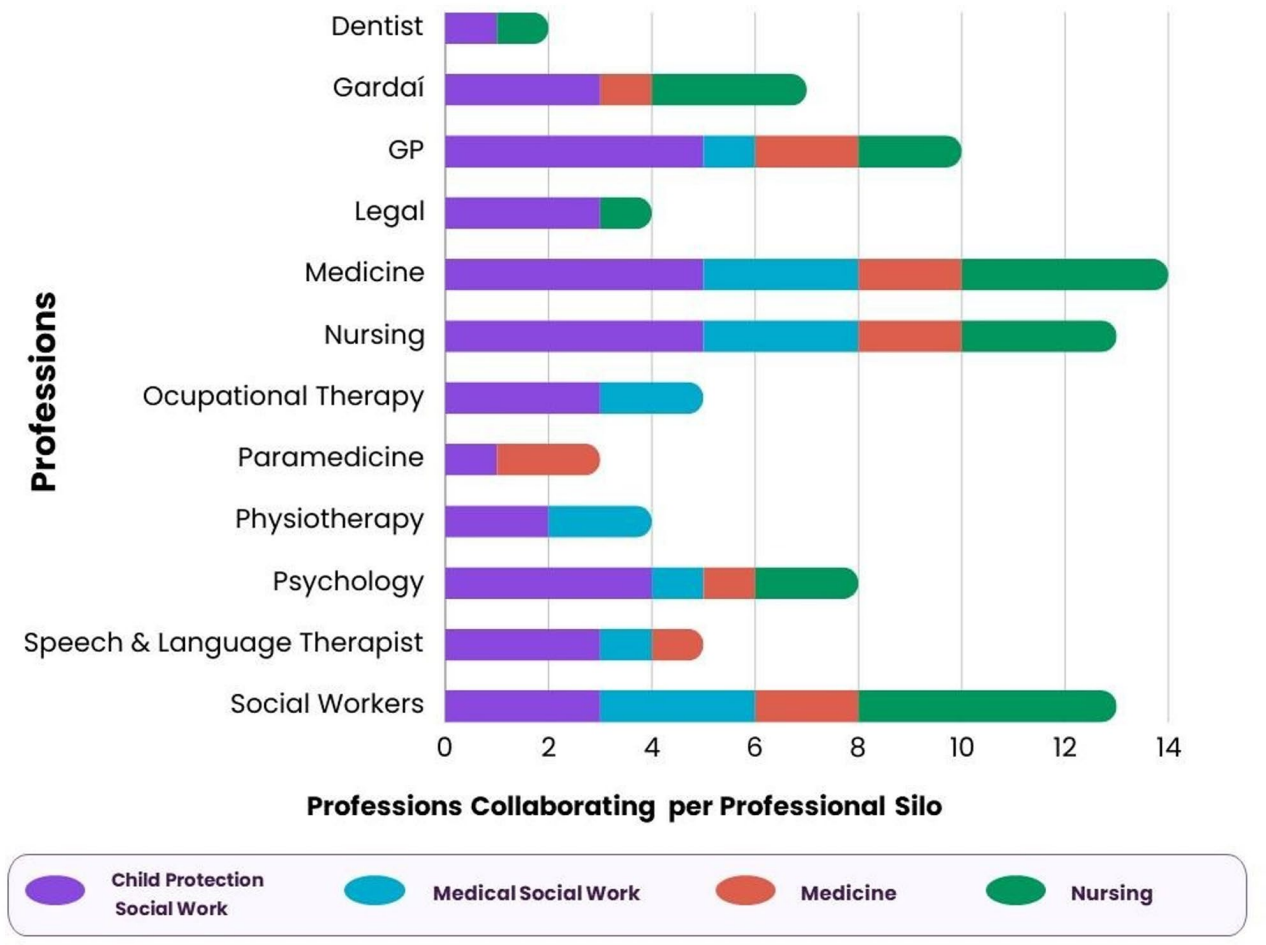
Interprofessional experience three months prior to training intervention

Additionally, participants were asked to list the number of other HCPs they engaged or collaborated with while managing suspected child safeguarding cases in the three months prior to taking part in the training intervention (see Fig. 4). The Túsla social workers and the pediatric nurses engaged with more professionals than the medical social workers and the medical doctors.

### Qualitative themes

Using the landscapes of practice principles of engagement, imagination, and alignment as a theoretical lens, we generated three key themes from our participant focus groups and interviews. These themes include: [1] collaborative learning [2], the medium of language and [3] creating a safe space; representing our participants’ experiences in navigating interprofessional learning in newly formed teams during simulated child safeguarding scenarios in the emergency department. The themes both facilitated and influenced each other, i.e. creating a safe space enhanced collaborative learning among the interprofessional teams and sharing the nuances of each discipline’s lexicons contributed to both psychological safety and collaborative learning. Combining the learnings from this course enabled knowledge translation and integration into the workplace, namely enhanced communication skills with both colleagues and patients, and a deeper understanding of the multi-professional approach required for effective child safeguarding practices. Additional supportive quotes are provided in Appendix D.

#### Collaborative learning

Collaborative learning encompassed a wide range of experiences, with participants becoming more aware of the collective responsibility for safeguarding children and the importance of understanding each other’s professional roles. They reflected on how teamwork is essential in real-world situations where child-centred care is the priority. As one participant explained, *“The reality is that child protection is everyone’s responsibility*,* and it doesn’t just happen when the social worker is in the room”* (P08, MSW).

Many participants acknowledged gaps in their knowledge of other professionals’ roles, recognising that siloed working had limited their perspective. Interprofessional training was seen as breaking down barriers and fostering respect. A nurse described, *“But when you actually open those doors and you get a glimpse inside of what the other service provides and how the other professionals work*,* I think it kind of breaks down those walls and makes it more of a team and interprofessional effort than just me versus you”* (P18, N). Another nurse reflected on how role clarity shifted assumptions: *“…how beneficial it is to know what other people’s roles are and what they can and cannot do…so I*,* as a nurse will know what exactly my social worker is going to help with and what Túsla will do in a case*,* for example. I probably didn’t know that much*,* If I’m being totally honest…oh*,* I just handed it over to the social worker*,* but that’s not the case*,* you know?”* (P11, N).

Participants highlighted the value of cooperation between hospital-based and community-based teams, recognising how integration strengthened continuity of care. One team social worker noted, *“I have the height of respect for the medical teams and trust their judgement completely. And it’s just having it joined up between ourselves*,* the community-based teams and the medical teams…it’s lovely too*,* the joint approach*,* (be)cause it makes it more real…It’s like reading a book and acting out a role—it’s just so much more powerful”* (P17, TSW).

Beyond procedural knowledge, participants valued the lived experience of colleagues and the opportunity to share struggles in a supportive environment. A doctor reflected, *“Actually getting to engage and being on the same course as people from the different disciplines just changed everything cuz you could actually discuss it with them. Things are so much different when you talk to someone with life experience and who’s doing the job*,* than just reading about it in a book or maybe hearing from say one expert*,* like one lecture*,* just actually getting to chat things through”* (P33, Dr). Collaborative learning also eased the burden of responsibility by normalising shared accountability. As one doctor explained, *“So I think that kind of eases things as well in my mind a little bit when*,* you know that the other professionals on the team are sending off their referrals as well to add to yours and fill the picture”* (P24, Dr).

Finally, participants described how interprofessional exchanges challenged assumptions and reduced tensions. A social worker reflected, *“Seeing what the doctors are having to deal with… I can understand why they’re focused on the medical bit…it was a bit of a reality check…This is a sick kid and needs a doctor to look after them”* (P04, MSW). A doctor echoed this from her perspective: *“…The social worker was like*,* wow*,* I didn’t realise you done that much work or you spent that much time… then we were explaining*,* myself and the nurse*,* [how] a case would often take a few hours in A and E before you’d be contacting Túsla. And they were very surprised at that”* (P33, Dr).

Together, these reflections illustrate how collaborative learning transformed participants’ practice. By breaking down silos, clarifying roles, and fostering empathy, the experience reinforced that safeguarding is not the responsibility of one profession alone but a coordinated, shared enterprise.

#### The medium of language

Shared learning across medicine, nursing, and social work highlighted the importance of communication strategies and the use of shared language. Participants consistently identified this as a key takeaway, both for working across professional boundaries and for interactions with parents. A simple telephone exercise revealed discipline-specific tools and mnemonics that many had not previously encountered. For example, “Signs of Safety” is widely used in social work referrals, while “SBAR” (Situation, Background, Assessment, Recommendation) is a common handover protocol in medicine. These tools were seen to enhance understanding of both immediate situations and the broader context of non-accidental injuries. As one participant explained, *“We do a lot of referrals to Túsla but we don’t have Signs of Safety that they use as their checklist*,* you know*,* from the referrals they are getting from us so then it’s difficult. There’s a loophole there for not putting the correct information in… we don’t have that piece of the jigsaw*,* that they are using as their tool”* (P09, MSW).

Reflecting on these tools in a safe learning environment gave professionals confidence to ask more relevant questions in practice. One participant described, *“I had a phone conversation the other day with a doctor who said something that I didn’t know*,* but I just asked him about it and he was like*,* oh*,* sorry.*,* and explained it. And it’s just nice when you have that respect for each other. And it’s very easy to just be open and be aware that*,* we may not know the same terms …. And so I felt like that was highlighted in that simulation”* (P31, MSW). This training provided clarity and permission to acknowledge knowledge gaps openly.

Observing others in simulated scenarios also offered new insights into communication styles. A team social worker noted, *“Certain words*,* certain things that I would have picked up on from maybe two people in particular*,* a nurse and a social worker in that scenario and it was the way that they brought the information to the client’s attention but in a very non-threatening way… it was lovely to observe”* (P14, TSW).

Participants valued learning specific phrasing that could reassure parents. A doctor reflected, *“I learned a few very crisp lines from that discussion…. We try to stay empathic*,* but their tone and everything well that was a big learning point for me…. I learned a few new words that I should say to parents to make them more comfortable”* (P13, Dr). These exchanges across professions were seen as particularly helpful. Another doctor described, *“During the break after my simulation*,* I talked to the social worker… and what they would do is sit down and give a warning shot…they would just lay down everything on the table without jargon and tell them ‘this is what is happening’. And the greatest insight that I got from that was that yes*,* you’re telling the parent that ‘this is what is happening and we are not blaming you*,* but if this continues*,* this is what will happen to your child’…And I will be using that now in my profession”* (P24, Dr).

Faculty input during debriefing sessions further reinforced the importance of clarity. One participant recalled, *“So ‘the main thing is to say the main thing’*,* is what he said. And I think that really resonated with me… that it’s important to make sure that you keep your message concise and that everyone can pick up what you are saying*,* ‘cuz what you say and how people perceive it can be two different things”* (P34, Dr).

Finally, the child’s perspective was incorporated through role play facilitated by EPIC, ensuring that the child’s voice was not overlooked. As one participant reflected, *“I thought it was really good to have the representative from EPIC…. [It made us] think about how we talk to children*,* how we talk to them about what’s happening. And even when we try not to use jargon*,* we probably are. So I think it was really powerful to just hear that voice”* (P08, MSW).

Generally, the medium of language was described as transformative, equipping participants with practical tools, confidence, and awareness to communicate more effectively across disciplines and with families.

#### Creating a safe space

Participants entered the training from diverse professional backgrounds and with varying levels of experience in both day-to-day practice and child safeguarding expertise. While the course was promoted as simulation-based, some had greater familiarity with this educational technique than others. Most had already completed the mandatory online *Children First* safeguarding training, making the establishment of psychological safety a critical element for success. As one participant reflected, *“We’re all professionals that were coming into something new that none of us had actually done anything like this previously. So we were all on the same level”* (P31, MSW).

Deliberate strategies were used to create a safe learning environment where participants felt comfortable speaking up and sharing experiences. One doctor explained, *“In some courses they will say so who’s interested to speak… you have to put your hand up. And for me*,* I think it’s intimidating*,* especially I’m from [country] so English is not actually my first language… So I liked being addressed by name*,* it was very encouraging”* (P01, Dr).

The training also addressed challenges of hierarchy and power dynamics in healthcare. A team social worker observed, *“There’s a total flattening of hierarchy*,* which for me is not an issue…but I remember working in [setting] and there was very much an issue between different professions but there’s none of that [here]. It’s a total focus on the child and the presentation. And it’s really nice to see the respect people have for each other”* (P17, TSW).

Participants described feeling valued and included regardless of role or experience, which fostered openness and collaboration. As one doctor noted, *“…Inclusion is one of the pillars of psychological safety and I really saw that shine throughout the simulations*,* and where everybody was given a chance to talk*,* to bring their ideas onto the table. Everybody had a chance to input*,* and to get something out of it. So that was really good. And it was psychologically safe. Everybody felt that they could do that”* (P24, Dr).

Relationship-building was facilitated through icebreakers and structured group activities. A doctor recalled, *“I really liked the comic thing [teambuilding exercise] because everyone was having fun and we managed to break the ice with everyone. So when we went into smaller groups*,* it was easier. Like you could ease into it”* (P01, Dr). Small group allocations were particularly valued, supporting both collaborative learning and psychological safety. A nurse explained, *“Breaking off into small groups for tasks as well as being in the same group the first day*,* and the second day was probably beneficial because I found on the second day we were all chatting about what we were doing at the weekend. And you’ve only met these people!”* (P03, N).

Informal interactions also played a role in building trust and future collaboration. One participant reflected, *“But I think the breakout*,* the lunch and just having that informal 15 minutes with someone*,* again*,* just builds up that relationship and that trust and that ability to be able to say*,* oh actually if you ever wanted more information on that*,* give me a call or vice versa”* (P08, MSW).

In summary, participants described the training environment as inclusive, respectful, and psychologically safe. Strategies such as flattening hierarchy, encouraging participation, and fostering informal connections were seen as essential in creating a supportive space for interprofessional learning.

## Discussion

In summary, our simulation-based child safeguarding intervention impacted participants’ learning and professional practice in various ways. First, our findings suggest that the course experience catalyzed a shift from uncertainty towards greater clarity in terms of increasing recognition that child safeguarding is a shared endeavor rather than an individual burden. Participants gained confidence in their decision-making to recognize child safeguarding concerns, despite complex clinical presentations and challenging conversations. Multiple quotes demonstrate how interprofessional dialogue was not only valued but also transformative in broadening professional perspectives and aligning child-centered needs across different sectors. The course also promoted enhanced child safeguarding lexicon, supporting interprofessional communications and shared vocabulary for future practice. Psychological safety was not only a feature of the training environment but also a facilitating factor, enabling learners to practice challenging decisions, learn from each other’s mistakes, all while developing trust across professions.

The three principles of LoP theory [23] framed our most important findings as established mechanisms of IPE, rather than incidental experiences:

1. Engagement- Our participants actively engaged with each other’s perspectives, breaking down siloes and recognizing shared responsibility for safeguarding.
2. Imagination- Simulated cases afforded participants opportunities to imagine themselves in other roles, developing empathy for colleagues and adopting new approaches to communicate with parents and children.
3. Alignment- Shared language, clarified roles and collaborative decision-making helped participants reach a collective child-centered goal.

We can further interpret our findings through the perspectives proposed by Hean et al. [18]. Participants described breaking down professional silos that hampered communication, recognizing safeguarding as a collective responsibility. These findings resonate with social capital theory, in which IPE fosters trusting relationships across professional boundaries [18]. The sense of psychological safety, equality, and mutual respect reported by course participants in our study reflects the conditions outlined in contact theory, where equal-status interaction under a shared goal (child safeguarding) flattens hierarchy and encourages honesty. Finally, the simulated scenarios, in which participants observed and adopted colleagues’ language, tools, and communication strategies, illustrated the principles of CoP, with learners moving from passive observation to active participation in authentic interprofessional practice. Taken together, these theoretical insights provide a robust explanation of why the training not only enhanced knowledge and confidence, but also transformed how participants collaborated, communicated, and aligned for the shared goal of safeguarding children.

Clinically, uncertainty exists when deciding whether to report suspected abuse, and cases of child abuse and neglect are not always obvious, with a recent Irish study showing that over 90% of clinical examinations often appear normal [31]. These ‘normal’ examinations can cause delayed disclosures and systemic issues with accessing support, further adding to the emotional burden on staff [31]. Our course gave professionals the tools and opportunities to apply them in exactly these conditions, reinforcing psychological safety as a prerequisite for full interprofessional participation. Effective psychologically safe learning environments consist of three elements: (a) mistakes are welcomed, (b) facilitators role model integrity, honesty, curiosity etc. to facilitate positive learning experiences, and (c) foundational activities are layered into the training to build expectations and trust before high-stakes simulation is expected [32].

While the main focus of our study was post-registration professionals, our findings align well with a realist review that highlights interprofessional learning for undergraduate healthcare students. Maddock et al. [33] outline that interdependence and embodiment are essential elements for achieving effective IPE outcomes. Interdependence means that learners must genuinely need one another’s contributions, especially in sensitive contexts. Merely being present is insufficient; the tasks must require skills and knowledge from more than one profession to succeed [33]. Our participants valued and relied upon one another’s contributions, reframing child safeguarding as a shared interdependent process, often requiring different pieces of information from police, paramedics, medical and social work professionals. Embodiment then refers to creating immersive, authentic scenarios to help learners feel the realities of interprofessional practice [33]. Engaging trained SPs to act as parents in our course reinforced the emotional aspects of the simulated scenarios, creating an immersive experience for our participants dealing with challenging cases. This helped learners empathize with both colleagues and parents, adopt new communication practices, and rehearse responses to sensitive safeguarding scenarios in a safe, supportive environment. Maddock et al. [33] also recognized the importance of involving skilled facilitators in sensitive IPE contexts, as well as building in time for reflection, which were key strengths of our study.

### Strengths and limitations

To the best of our knowledge, few if any studies have explored interprofessional simulation-based education with child safeguarding professionals in the emergency department context. By including professionals from medicine, nursing, and social work with varying levels of experience mirrors the real-world composition and complexity of safeguarding teams, making our findings directly relevant to practice. Using simulation-based education proved innovative, as most post-registration child safeguarding education in Ireland consists of asynchronous self-paced e-learning modules. In addition, the broad perspectives and expertise within our research team also added to the robustness of this study, as many members of the research team experienced these child safeguarding issues firsthand. We recognize that our study was conducted in a single site and our sample was both purposive and relatively small. Participants did not represent all professionals working in the chain of child safeguarding meaning we may have missed some important perspectives, although the multiple course iterations, multi-method approach, and rich data help counter this limitation. Future course iterations could adapt to different contexts and include different professional perspectives e.g. teachers, lawyers, dentists, community services.

### Implications for practice

Child safeguarding demands continuous interprofessional collaboration and teamwork to ensure children at risk of abuse and neglect don’t fall through the cracks of a fractured system. Our study contributes important pragmatic learnings as implications for future simulation-based approaches to improve interprofessional collaborative practice in this context. Based on our findings and our knowledge of simulation-based education, we propose five design principles to guide others seeking to develop similar courses:

**Table:** 

1. Design scenarios that reflect authentic nuance and complexity to mirror the real cases professionals encounter in practice, by fostering cross-professional interdependence and creating genuine opportunities for interprofessional learning through participation, observation and facilitated debriefings.
2. Recruit course participants with diverse experience levels whenever possible to foster cross-professional learning.
3. Embed psychological safety from the beginning of healthcare professional training and attend to it through all course phases.
4. Foster the use common safeguarding vocabulary throughout the course, since developing a common language to communicate across professional boundaries was transformative for our participants.
5. Integrate the child’s voice to anchor the safeguarding focus on the child.

These learnings about interprofessional course design are relevant for the workplace, and ideally should be replicated in team-based training. Continuous professional development could include follow-up case-based reviews or team reflective huddles to reinforce skills learned during the simulations, feeding into systems level or quality improvement initiatives.

## Conclusion

Our study shows how a co-designed interprofessional simulation-based child safeguarding course facilitated space to renegotiate safeguarding as a shared, interdependent responsibility, rather than an individual burden. Participants suggest that working with authentic, emotionally charged scenarios in a psychologically safe environment enabled them to sit with uncertainty, experiment with reporting decisions, and develop a more shared safeguarding lexicon across professional boundaries. The design principles we offer may resonate with educators and services in similar settings, seeking to co-create interprofessional child safeguarding education that foregrounds psychological safety, authentic collaboration and the child’s voice. We hope that this study can inform future inquiry into how simulation shapes collaborative safeguarding practice over time.

## Supplementary Information

Supplementary Material 1.

Supplementary Material 2.

Supplementary Material 3.

Supplementary Material 4.

## Data Availability

The datasets generated and/or analysed during the current study are not publicly available but are available from the corresponding author on reasonable request.
