# Supplementary material for: The CHILD safeguarding simulation study: Co-designed cHild-centred Interprofessional Learning through Dialogue for healthcare professionals
Source: Adv Simul (Lond). 2026 Jan 9;11:7. doi: 10.1186/s41077-025-00403-w (PMC12888331; doi:10.1186/s41077-025-00403-w)
Supplement: Supplementary file 2 — Supplementary Material 2. [file 41077_2025_403_MOESM2_ESM.docx]

**Topic Guide**

**Opening Comments**

We’ve invited you to participate in this interview since we are interested in hearing what you took away from the course and what helped you learn. Also, we are keen to hear your perspective on how we can make the course better.

**General opening questions about course**

1. Now that you have had time to reflect on the course, would you share your main takeaways?

**Learning outcomes: (Self)**

1. What supported your learning during the course?
2. How would you now describe your role in keeping children safe?

**Learning outcomes (Others)**

1. What helped you engage with other professionals both during the simulations and group discussions to create a more collaborative environment?
2. What, if anything, did you learn from other professionals? (e.g. nurses, social workers, physicians)

**Learning environment:**

1. There may have been times during the course where you felt more or less willing to share your thinking, especially if you were unsure if you were right about something. What were these moments for you?

- Might you give an example? What helped you contribute?

**Learning outcomes (System)**

1. How has this course made you think differently about child safeguarding?
2. How would these learnings improve your profession?

**Reflection**

1. If you had a magic wand, what would you change about the course?
2. What other topics, if any, should have we covered on this course?

**Closing**:

- Summarise key points of the discussion
- What have we missed that you feel is valuable for this discussion?

**Probes:**

Examples of follow-up questions

- Please tell me more.
- Could you expand on that?
- Might you provide an example?
- How come?
- What makes that aspect a challenge?
