# Supplementary material for: The CHILD safeguarding simulation study: Co-designed cHild-centred Interprofessional Learning through Dialogue for healthcare professionals
Source: Adv Simul (Lond). 2026 Jan 9;11:7. doi: 10.1186/s41077-025-00403-w (PMC12888331; doi:10.1186/s41077-025-00403-w)
Supplement: Supplementary file 3 — Supplementary Material 3. [file 41077_2025_403_MOESM3_ESM.docx]

Appendix A

| Tablex: Overview of the emergency department simulation scenarios developed for the interprofessional child safeguarding course | | | | |
| --- | --- | --- | --- | --- |
| Scenario | Scenario Aim(s) | Learning Objectives | Simulated Participant | |
|  |  |  | Role | Engagement |
| A | To demonstrate effective interprofessional team working to evaluate and manage a critically ill child with apparent non-accidental injury. | 1. Coordinate an interprofessional approach to evaluate and manage an acutely unwell child due to apparent non-accidental injury with parents present  2. Listen to the perspective of a first responder in cases of potential non-accidental injury  3. Demonstrate non-blaming empathic patient-centered communication when discussing concerns of non-accidental injury  4. Communicate effectively with relevant members of the health and social care team | Mother | Mother arrived home to the baby who was hysterical and could not be soothed. Baby was under the father’s care. She appears concerned and has very little eye contact with team. She gives one-worded answers to teams questions. She is concerned for her child’s wellbeing but is also worried that her children will be taken away from her. |
|  |  |  | Father | Father acts very defensively and answers questions posed by the team. Father continually demands to see the baby. He will not explain how the incident occurred without repeated questioning and until there is a successful communication strategy from the team. |
| B | To demonstrate inter-professional coordination and communication in instances of threatened patient elopement during an evaluation for non-accidental injury.    To demonstrate non-blaming empathic patient-centered communication in cases of potential non-accidental injury. | 1. Coordinate an inter-professional approach for a patient/family who would like to leave before investigations about possible non-accidental injury are complete  2. Collaborate within the team to communicate the need for additional evaluation (social work, additional investigations) before leaving the A&E  3. Demonstrate non-blaming empathic patient-centered communication when discussing concerns of non-accidental injury  4. Discuss legal requirements related to parental decision-making in instances of abuse (not wanting evaluation, wanting to defer evaluation, etc.) | Mother | Mother attended the hospital due to concerns about a possible ear infection since her daughter was pulling on her ear. She is concerned that the child’s symptoms are getting worse so she brought her in.    Mother can be occupied by her phone and is easily frustrated for no clear reason. Mother appears impatient and may drum her fingers or tap her foot a lot. She is very protective of her child and is dismissive of any suggestion that she is doing a bad job. She appears uneasy when being asked questions – and doesn’t answer what is asked instead diverts the topic.    She indicates that she is about to leave but does not actually leave at any point. |
| C | To demonstrate an approach to the assessment of potential child sexual abuse. | 1. Coordinate an interprofessional approach to evaluate and manage a suspected case of child sexual abuse with parent.  2. Discuss procedural requirements relating to a suspected case of sexual abuse.  3. Collaborate with team to determine a plan to manage this patient. | Mother | Mother presents to A&E with daughter concerned that her daughter’s behavior is different. It is a Sunday afternoon and she has collected her child from her father’s, where she has spent the weekend with his new family. She is worried that someone has touched her inappropriately and wants her checked out to tell if someone is abusing her. Child is not acting like herself, quieter, and Mum was concerned. Mum noted redness in pelvic area.  Mother is agitated and concerned for child's wellbeing. She wants answers, is very responsive and cooperative, and is seeking guidance from the team |

**Basic Debriefing Script*** *(modified from Eppich and Cheng, 2015)*

| **Before the simulation** | |
| --- | --- |
| 1) Introductions  2) Review ground rules  a. *“Everyone is capable, does their best, and wants to improve”*  b. *“We don’t expect perfection, our goal is learning and improvement”*  c. *“Everyone has something to contribute”*  d. Confidentiality: *“Take away learnings, not individual performances”*  e. *“People act differently in simulation than in real life—focus on the clinical issues*  3) *“During the debriefing we will explore how you cared for the patient, how you worked together and aspects of both that worked for you and those you would change.”* | |
| **Debriefing** | |
| **Setting the stage** | “We will spend about [X] minutes debriefing. I expect to hear from everyone and hope you share what was on your mind at various points during the case.” |
| **Reaction** | “How are you feeling right now?”  **OR**  “Initial reactions?” |
| **Description*** | “May I invite someone to give a **one line summary** of the case to make sure we are all on the same page? |
| **Analysis** | “From your perspective, what worked well **and why**?”  “What would you change next time, **and why**?  Consider probing / facilitating focused discussion around key topics, and providing relevant feedback/information |
| ***Any other burning issues before we start winding down?*** | |
| **Summary** | *What are you taking away from this experience?* |

*Educator can state main issues for participants, especially if time is limited

From *PEARLS Scripted Debriefing Tool (*Walter Eppich, MD, PhD, Adam Cheng, MD)

| **Pairing your point of view with a question to get trainees’ perspective**  ***Step 1: Previewing:*** Frame the discussion, switch gears to a new topic    ***“Let’s talk about….[topic]*** | | |
| --- | --- | --- |
| ***Step 2***  **Observation about the performance** | ***Step 3***  **Share your point of view about the performance** | ***Step 4***  **Solicit the learner perspective** |
| *I noticed that…*  *I saw that…/I didn’t see..*  *I heard you say…** | **Appreciation**  · *I liked that….*  · *I thought that was interesting/ fascinating…*  **Appreciation or concern**  · *I was thinking…*  · *That makes me think that…*  · *I had the impression that…*  · *It seemed to me that…*  **Concern**  · *I was wishing that*  · *I felt uncomfortable because…*  · *I was worried/concerned…* | · *How do you all see it?*  · *I wonder what your thoughts were at the time?*  · *What was going through your mind?*  · *What were your priorities at the time?*  · *How did the team get organized?* |
