# Supplementary material for: The CHILD safeguarding simulation study: Co-designed cHild-centred Interprofessional Learning through Dialogue for healthcare professionals
Source: Adv Simul (Lond). 2026 Jan 9;11:7. doi: 10.1186/s41077-025-00403-w (PMC12888331; doi:10.1186/s41077-025-00403-w)
Supplement: Supplementary file 4 — Supplementary Material 4. [file 41077_2025_403_MOESM4_ESM.docx]

| **Theme** | **Participant quote (ID, role)** | **Illustrative point** |
| --- | --- | --- |
| Collaborative learning | “Actually getting to engage and being on the same course as people from the different disciplines just changed everything cuz you could actually discuss it with them. Things are so much different when you talk to someone with life experience and who's doing the job, than just reading about it in a book or maybe hearing from say one expert, like one lecture, just actually getting to chat things through” (P33, Dr) | Value of learning directly with and from other disciplines |
| Collaborative learning | “I have the height of respect for the medical teams and trust their judgement completely. And it’s just having it joined up between ourselves, the community-based teams and the medical teams…it’s lovely too, the joint approach, (be)cause it makes it more real…It’s like reading a book and acting out a role- it’s just so much more powerful” (P17, TSW) | Appreciation of joined up, hospital-community approach |
| Collaborative learning | “…how beneficial it is to know what other people's roles are and what they can and cannot do…so I, as a nurse will know what exactly my social worker is going to help with and what Túsla [will do in a case, for example. I probably didn't know that much, If I'm being totally honest…oh, I just handed it over to the social worker, but that’s not the case, you know?” (P11, N) | Increased role clarity and changed assumptions about “handing over” |
| The medium of language | “We do a lot of referrals to Túsla but we don't have Signs of Safety that they use as their checklist, you know, from the referrals they are getting from us so then it's difficult. There's a loophole there for not putting the correct information in… we don't have that piece of the jigsaw, that they are using as their tool” (P09, MSW) | Recognition of missing shared tools and their impact on information quality |
| The medium of language | “Certain words, certain things that I would have picked up on from maybe two people in particular, a nurse and a social worker in that scenario and it was the way that they brought the information to the client’s attention but in a very non-threatening way… it was lovely to observe” (P14, TSW) | Learning specific phrasing and tone from observing others |
| The medium of language | “I learned a few very crisp lines from that discussion.... We try to stay empathic, but their tone and everything well that was a big learning point for me…. I learned a few new words that I should say to parents to make them more comfortable” (P13, Dr) | Acquisition of practical language for use with parents |
| The medium of language | “So ‘the main thing is to say the main thing’, is what he said. And I think that really resonated with me… that it's important to make sure that you keep your message concise and that everyone can pick up what you are saying, ‘cuz what you say and how people perceive it can be two different things” (P34, Dr) | Emphasis on concise, clear messaging |
| The medium of language | “I thought it was really good to have the representative from EPIC…. [It made us] think about how we talk to children, how we talk to them about what’s happening. And even when we try not to use jargon, we probably are. So I think it was really powerful to just hear that voice” (P08, MSW) | Influence of the child’s perspective on communication awareness |
| Creating a safe space | “We're all professionals that were coming into something new that none of us had actually done anything like this previously. So we were all on the same level” (P31, MSW) | Perception of equal footing in a new learning format |
| Creating a safe space | “In some courses they will say so who's interested to speak... you have to put your hand up. And for me, I think it's intimidating, especially I'm from [country] so English is not actually my first language... So I liked being addressed by name, it was very encouraging" (P01, Dr) | Impact of facilitator strategies on willingness to participate |
| Creating a safe space | “There’s a total flattening of hierarchy, which for me is not an issue…but I remember working in [setting] and there was very much an issue between different professions but there’s none of that [here]. It’s a total focus on the child and the presentation. And it’s really nice to see the respect people have for each other” (P17, TSW) | Reduced hierarchy and focus on the child |
| Creating a safe space | “…Inclusion is one of the pillars of psychological safety and I really saw that shine throughout the simulations, and where everybody was given a chance to talk, to bring their ideas onto the table. Everybody had a chance to input, and to get something out of it. So that was really good. And it was psychologically safe. Everybody felt that they could do that” (P24, Dr) | Perceived inclusion and psychological safety |
| Creating a safe space | “I really liked the comic thing [teambuilding exercise] because everyone was having fun and we managed to break the ice with everyone. So when we went into smaller groups, it was easier. Like you could ease into it” (P01, Dr) | Role of team-building in easing participation |
| Creating a safe space | “Breaking off into small groups for tasks as well as being in the same group the first day, and the second day was probably beneficial because I found on the second day we were all chatting about what we were doing at the weekend. And you’ve only met these people!” (P3, N) | Small group continuity and relationship-building |
| Creating a safe space | “But I think the breakout, the lunch and just having that informal 15 minutes with someone, again, just builds up that relationship and that trust and that ability to be able to say, oh actually if you ever wanted more information on that, give me a call or vice versa” (P08, MSW) | Informal interactions supporting future collaboration |

Appendix D Supplemental quotes
